# Occupational safety and health, green chemistry, and sustainability: a review of areas of convergence

**DOI:** 10.1186/1476-069X-12-31

**Published:** 2013-04-15

**Authors:** Paul A Schulte, Lauralynn T McKernan, Donna S Heidel, Andrea H Okun, Gary Scott Dotson, Thomas J Lentz, Charles L Geraci, Pamela E Heckel, Christine M Branche

**Affiliations:** 1 National Institute for Occupational Safety and Health, Centers for Disease Control and Prevention, Cincinnati, OH, USA; 2 Bureau Veritas, Edison, NJ, USA

**Keywords:** Environmental health, Life cycle analysis, Workers, Alternatives, Nanomaterials, 1-Bromopropane

## Abstract

With increasing numbers and quantities of chemicals in commerce and use, scientific attention continues to focus on the environmental and public health consequences of chemical production processes and exposures. Concerns about environmental stewardship have been gaining broader traction through emphases on sustainability and “green chemistry” principles. Occupational safety and health has not been fully promoted as a component of environmental sustainability. However, there is a natural convergence of green chemistry/sustainability and occupational safety and health efforts. Addressing both together can have a synergistic effect. Failure to promote this convergence could lead to increasing worker hazards and lack of support for sustainability efforts. The National Institute for Occupational Safety and Health has made a concerted effort involving multiple stakeholders to anticipate and identify potential hazards associated with sustainable practices and green jobs for workers. Examples of potential hazards are presented in case studies with suggested solutions such as implementing the hierarchy of controls and prevention through design principles in green chemistry and green building practices. Practical considerations and strategies for green chemistry, and environmental stewardship could benefit from the incorporation of occupational safety and health concepts which in turn protect affected workers.

## Background

**Figure 1:**
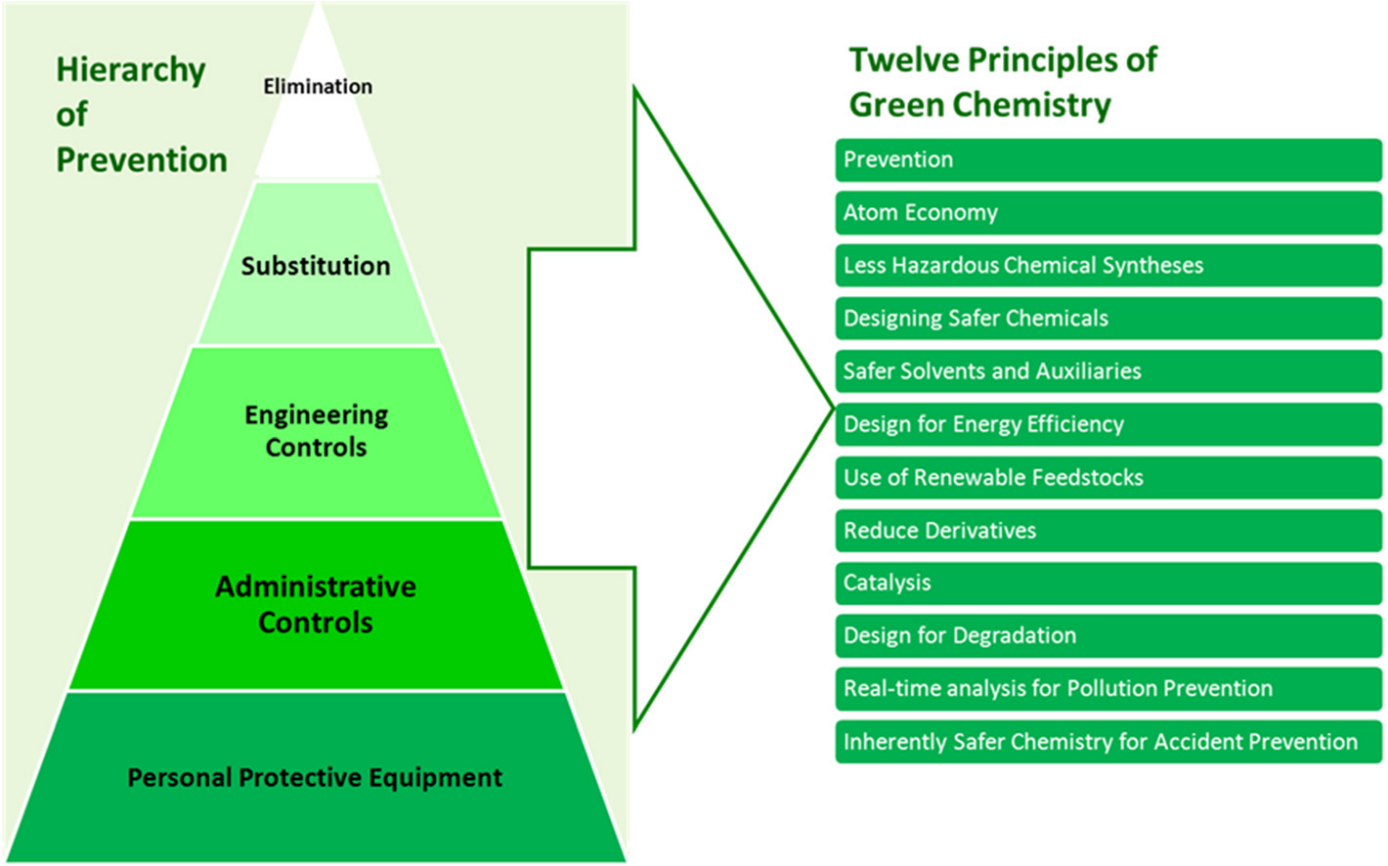
Green Chemistry and Application of Hierarchy of Controls.

Integration of occupational safety and health (OSH) with sustainability and green chemistry practices is essential to the effective realization of all of these endeavors. Sustainability has a plurality of definitions [1]. In the ecological area “sustainability” calls for policies and strategies that meet societies’ present needs without compromising the ability of future generations to meet their own needs [2]. Green chemistry is a suite of 12 enabling principles intended to lead to chemical products and processes that are more efficient, use less toxic materials, and produce less waste in the environment (Figure 1) [3]. If green chemistry is applied and workers are not considered, there is the likelihood that workers could be harmed and the full investment in green chemistry will not be realized. There is increasing scientific understanding of the human and environmental health consequences of chemicals and the energy demands associated with chemical processing. National and international regulatory policies are inefficient in keeping up with the myriad of chemicals used in commerce today [4,5]. The U.S. Environmental Protection Agency (EPA) uses the US Toxic Substances Control Act (TSCA) to monitor approximately 80,000 chemicals. In the European Union, the 2006 regulation on the Registration, Evaluation, Authorization and Restriction of Chemicals (REACH) requires manufacturers to provide detailed information on compounds that are manufactured, marketed, or imported [6]. Workers have always been affected by chemical exposures. The history of occupational safety and health has been punctuated by research investigating the impact of chemical exposures on workers and by regulatory efforts for chemicals risk management. As society moves forward to balance economic necessity and industrial efficiency with potential environmental consequences, the implication for worker safety and health needs to be considered. Considering OSH when exploring green chemistry options will reduce the likelihood of unwarranted consequences for workers and the need to revise approaches to sustainability. The case can be made that existing corporate occupational safety and health risk management programs should now be extended to include the principles of green chemistry.

## Green chemistry and sustainability

The 12 principles of green chemistry can be roughly organized into two major categories: those related to reducing energy usage and waste materials, and those related to producing or utilizing safer products and processes [7]. While the application of these principles will lead to less energy consumption and the reduction of waste material put into the environment, these principles could also aid in protecting and improving worker safety and health [8,9].

When worker hazards and risks are considered during design or re-design of production processes in accordance with green principles, health gains, environmental benefits, and cost savings can be maximized. Similarly, public policies designed to promote green chemistry technologies can promote worker health by including occupational safety and health criteria.

The commonalities of environmental sustainability and occupational safety and health have been widely acknowledged. In fact some investigators “. . . suggest using safety as an entry point for operationalizing sustainability for an organization, with a dual emphasis focusing equally on human benefits and the business case in achieving this grander conceptualization of sustainability” [10]. In many cases environmental sustainability and occupational safety and health are impacted by the same factors. Some researchers have proposed including safety within a “Safe–Sustainability Continuum” where commitment to safety serves as a starting point toward achieving sustainable business practices [10,11].

Green chemistry can advance environmental sustainability by informing the design of molecules, manufacturing processes, and products in ways that conserve resources, use less energy, eliminate pollution, and protect human health. This approach has been expressed under initiatives identified as “green.” While endeavors that employ green chemistry have been heartily supported by the occupational safety and health community [7-10], the opportunity to fully incorporate health and safety into the sustainability paradigm has not yet been realized.

Processes and products that contribute to outcomes of sustainability, energy saving, and use of renewable resources can still have significant toxicological and physical hazards (Table 1) [12-20]. There are many examples where workers involved with processes that are considered “green” or renewable energy have significant risk for exposure to toxic substances. For example, during manufacturing of thin-film photovoltaics, exposure to silane is of significant concern as it is pyrophoric as well as an irritant for the respiratory tract and skin [12]. During electronic-waste recycling, workers can be exposed to rare earth elements, lead, mercury and other heavy metals [13,14]. It is most often workers who will be exposed to these hazards directly by handling raw material during production, packaging, and transport, during product use, or during eventual disposal or recycling of products at the end of their useful life. Conversely, the products and outputs of processes that utilize green chemistry principles could be beneficial to workers when occupational safety and health are considered. For example, reducing the amount of toxic intermediates in chemical production can minimize worker exposure risks. Alternatively, making safer products for consumers may be good for workers who use consumer products in their jobs. For example, workers employing less toxic cleaning products will likely experience better indoor air quality with reduced irritation for both workers and occupants.

**Table 1:** Examples of chemical hazards associated with green products and processes

**Process/product**	**Chemical agent**	**Effect/target organ**	**References**
Thin-film Photovoltaics			
	Silane	Pyrophoric, Irritant, Respiratory Tract, Skin	[12]
	Hydrogen sulfide	Acute, Irritant, Central Nervous System	[12]
E-waste Recycling			
	Lead	Cardiovascular, gastrointestinal effects	[13]
	Mercury	Gastrointestinal effects, Neurotoxicant, Nephrotoxicant	[13,14]
Weatherization			
	Isocyanates (Spray Polyurethane Foam)	Irritant, Respiratory Tract, Sensitizer	[15,16]
Geothermal Energy			
	Hydrogen Sulfide	Acute, metabolic disorder	[17,18]
	Mercury	Gastrointestinal effects, Neurotoxicant, Nephrotoxicant	[19]
Solvent Replacement			
	1-Bromopropane	Cancer, hematopoietic effects; Hepatotoxicant Neurotoxicant	[20]

## Green chemistry and toxicology

A fundamental aspect of green chemistry is the identification of and reduction of toxic effects of chemicals through the design of safer products [3]. Historically, the toxic effects of new chemicals and materials did not receive much consideration in the design process. Now, the link between green chemistry and toxicology is becoming a knowledge objective [21,22]. When new chemicals are designed to reduce the intrinsic toxicity, workers, as well as the general population will benefit. Green chemistry research can be advanced through the field of computational toxicology [21]. Computational toxicology uses computer models to predict the possible adverse health effects caused by chemicals and helps to prioritize the large number of chemicals that need testing. Additionally, advances in pharmacokinetics, High Throughput Screening (HTS), and toxicogenomics will allow for faster toxicity screening [21,23]. Designing substances where worker exposure is a possibility needs to include consideration of uncertainty around estimates because one chemical may be more green in one metric and less so in another [24]. Still, tools like EPA’s software called T.E.S.T (toxicity estimates software tool) can assist the chemical industry with development of green chemistry alternate products and processes by predicting toxicity of materials which could have an impact on workers’ safety and health as well [25].

## Green chemistry and application of hierarchy of controls

The most effective means of occupational risk mitigation and hazard control is through process changes that eliminate a hazard or substitutes a nonhazardous or less hazardous alternative, effectively designing-out or reducing worker exposures. A seven-year national initiative called Prevention through Design (PtD) sponsored by the National Institute for Occupational Safety and Health (NIOSH) promotes designing out hazards at all levels from molecules, tools, equipment, processes, structures [26]. PtD is consistent with the fundamental objective of green chemistry and focuses the process of design on worker protection. Additionally, a long used framework to control exposures in the occupational environment consists of substitution, isolation and ventilation followed by administrative programs [26]. This hierarchy of controls mitigates risks for workers by stepping through various options for control starting with elimination (Figure 1). As one moves downward through the hierarchy, the control approaches become less effective and less acceptable to workers, with the last resort being the utilization of personal protective equipment. The hierarchy of controls has been widely used and reiterated as an effective strategy for controlling workplace hazards [27-30].

As shown in Figure 1, the principles of green chemistry and occupational safety and health converge at every step of the hierarchy of controls, as both emphasize prevention and upstream solutions. Accordingly, occupational safety and health knowledge can inform the implementation of green chemistry and sustainability principles, if it is applied. The section below provides case studies within the framework of the hierarchy of controls to illustrate the nexus of these occupational safety and health, green chemistry, and sustainability fields.

### Elimination: solvent elimination

Elimination is the pinnacle of the hierarchy of controls because it completely removes the hazard from the work environment. The pharmaceutical industry has been employing sustainability principles, thereby improving productivity for several decades. The synthesis of Pregabalin, a pharmaceutical treatment for the nervous disorders such as epilepsy and social phobia, provides insight into how the elimination step of the hierarchy of controls and green chemistry suite work hand in hand. Originally, the Pregabalin synthesis process included the disposal of half the synthetic materials used to create the compound [31]. After pharmaceutical manufacturer Pfizer completed an enzymatic screen, the company identified two new enzymes which eliminated several chemical conversion steps, increased production speed, and reduced waste [31]. These improvements were based upon the E-factors of the compounds but had the added benefit of the removal of various solvents during the synthesis process. Not only did this improve efficiency, but it also reduced worker exposures during the disposal process.

### Substitution: alternative chemicals

Substitution is the second step of the hierarchy of controls as it replaces a substance with another with lower toxicity. Similarly, one of the 12 principles of green chemistry is to design safer chemicals and products to be fully effective yet have little or no toxicity [3]. A broad range of federal and local legislation and programs address the need to consider the green health and safety implications of current practices involving chemicals.

In fact, substitution which is the cornerstone of the comprehensive movement for alternatives assessment for chemicals is built on the hierarchy of controls which has substitution as a priority. In designing safer products however, new toxic effects can arise. For example, refractory ceramic fibers (RCFs) were engineered to replace many of the uses of asbestos, providing durable, lightweight, and highly-heat resistant insulation materials for multiple industrial and commercial applications. However, epidemiological studies with RCFs found that occupational exposures to these fibers are associated with adverse respiratory effects as well as skin and eye irritation [32]; experimental studies also indicated that exposures to RCFs may pose a carcinogenic risk based on the results of chronic inhalation studies which produced mesotheliomas in hamsters and lung cancer in rats [33,34]. Because the durability of the fibers affects their biopersistence and therefore toxicity after inhalation and deposition in the lungs and other tissues, manufacturers have explored options to develop newer synthetic vitreous fibers with chemical compositions for optimal biosolubility (i.e., making them more soluble and less biopersistent) [34,35]. Experimental inhalation studies of the dissolution of these less durable fibers indicate reduced potential for lung tissue toxicity. However, with the increased solubility and clearance of the newer fibers and their components, further evaluation may be necessary to determine whether toxicity is expressed elsewhere, e.g., possibly causing nephrotoxic effects.

Occupational safety and health should be considered in the implementation of green chemistry, thereby helping to avoid green chemistry decisions with unwanted consequences. For example, the principle for safer auxiliary substances includes solvents and it is likely that water will be one of the substitutes. However, the solvent properties of water are limited and higher pressure and temperatures are required which may create heat and burn hazards to workers. Another example involves the increased use of biomass and other raw materials which shifts the population at risk from fossil fuel workers to the agricultural workers. No new hazards are involved but there are increased exposures for a different worker subgroup. Pollution and accident prevention principles often involve keeping effluents and pollutants in a plant or factory. This may increase worker exposures and, in some cases, the risk of explosions and fires. The entire exposure scenario must be considered and occupational safety and health must be incorporated at every step.

The concept of “alternatives assessment” moves this concept forward by providing a process for identifying and comparing potential chemical and nonchemical alternatives to a chemical of concern to facilitate informed substitution [4,5]. Efforts to include occupational safety and health considerations into the alternatives assessment framework are underway.

### Engineering controls: transitioning machines and processes to green alternatives

Engineering controls are utilized to physically remove contaminants or exposure from the worker through isolation, local exhaust ventilation, engineering hoods or pressure differentials. The best engineering controls are those which are automatic and are reliable to reduce exposure, regardless of work practices employed.

**Figure 2:**
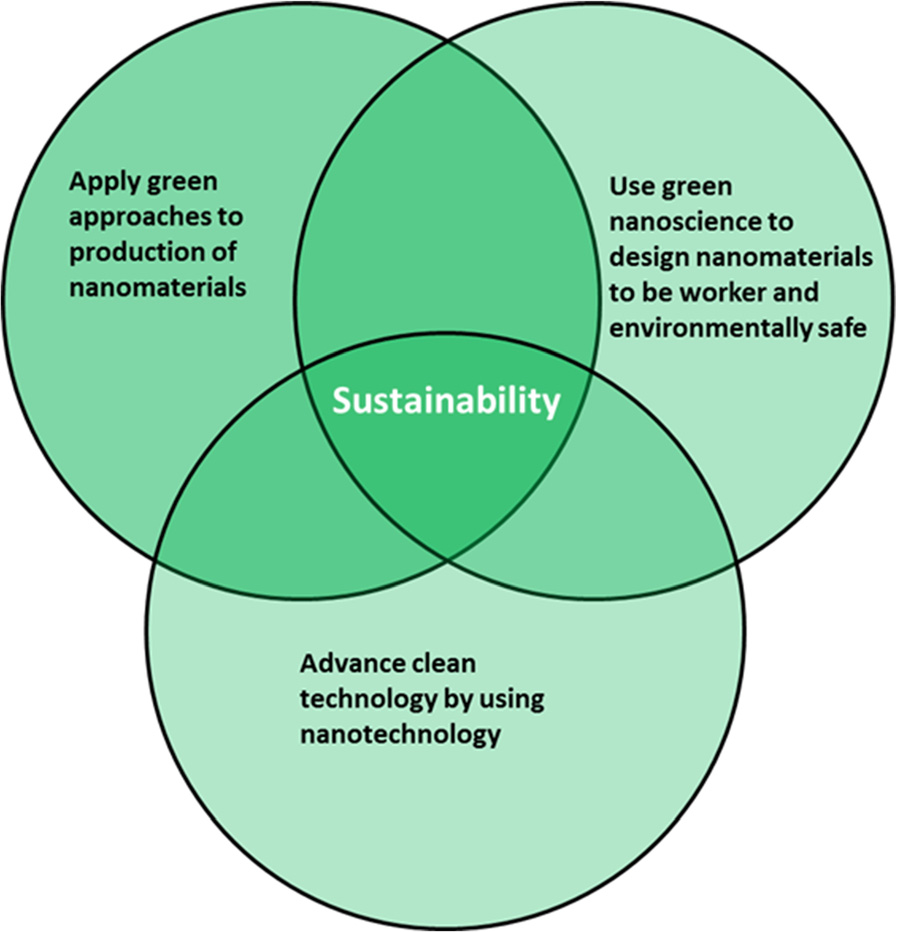
Nexus between nanotechnology and green approaches.

The field of engineered nanomaterials offers challenges and opportunities to eliminate exposures, especially when green chemistry and sustainability principles are applied in production. “While many nanoscale materials hold great promise of societal benefit making these materials comes at a high cost of energy, water, and the use of toxic chemicals [36].” Some of these nanomaterials may also be hazardous to workers and the environment [37,38]. There has been a swift expansion of nanomaterial research and their resultant products. As nanoscale inventions flourish, they require new approaches to upscale and deploy nanotechnology into the market. “There is an unusual opportunity to use science, engineering and policy knowledge to design novel products that are benign as possible to human and environmental health [39].” Nanotechnology also has the potential to serve as a means to achieve green technologies [40] and as such, the development of the green nanoscience concept has emerged [41,42]. Figure 2 shows the nexus between nanotechnology and green approaches. Green nanoscience is an approach that applies the 12 green chemistry principles to the design and production of nanomaterials [41,42].

Green nanoscience examples, such as the development of lower temperature and pressure fluid bed reactors, are beginning to emerge. Another example, using reactors to synthesize carbon nanofibers, a company was able to contain the process from catalyst introduction to product harvesting. Compared to working with dry powder and manual handling, the implementation of such engineering controls will likely result in reduced occupational exposures [43].

It has been advocated that if nanotechnology is to survive to maturity it must become green and facilitate green, otherwise it may be stunted by both real and perceived concern about harmful impacts on the environment and human health [40]. If worker health is threatened by nanotechnology it is unlikely that society will allow the technology to develop without economic ramifications. The responsible development of the technology requires attention to occupational safety and health. If green nanoscience is developed with attention to occupational safety and health hazards, the likelihood of further investment and expanded development will be increased.

### Administrative controls: LEED program for construction

Administrative controls can include work practices and administrative policies supplemented by exposure monitoring and medical surveillance that can result in a reduction of workplace exposure. Administrative controls are a critical component of where green chemistry and sustainability principles can overlap with occupational health and safety.

For example, the construction sector is both the place where many green innovations will be used and where workers performing many jobs labeled as green are at risk of injury, disease or death. A pivotal piece of the green building movement is the U.S. Green Building Council (USGBC) Leadership in Energy and Environmental Design (LEED) certification system. Use of the LEED Green Building Rating System is growing rapidly, with nearly 9 billion square feet of building space participating in the suite of rating systems and 1.6 million feet certifying per day around the world [44]. LEED is an internationally-recognized green building rating and certification system, providing building owners and operators with a framework for identifying and implementing practical and measurable green building design, construction, operations, and maintenance solutions. LEED promotes strategies intended to improve environmental and health performance of buildings using metrics such as energy savings, water efficiency, carbon dioxide (CO_2_) emissions reduction, improved indoor environmental quality, and stewardship of resources and sensitivity to their impacts [45].

USGBC defines green building as structures that have significantly reduced or eliminated negative impacts on the environment and the occupants [46]. The LEED system measures specific criteria related to sustainability and holds untapped promise to promote and protect occupational safety and health. LEED could be used as an administrative control if occupational safety and health are appropriately valued. Construction workers can be characterized as the earliest occupants in the initial lifecycle stage of a green building; these construction workers and others will also maintain, remodel, and decommission a green building throughout its lifecycle [47].

The proposed LEED V4 includes credits for the Avoidance of Chemicals of Concern [45]. These credits are intended to reduce the presence of potentially hazardous substances from the material supply chain. LEED points will be given if a construction project uses at least 20% (1 point) or 30% (2 points) by cost of all building products and materials that meet the requirements of European Commission Registration, Evaluation, Authorization, and Restriction of Chemicals (REACH) or third-party verified equivalent [6]. REACH aims to ensure the protection of human health and the environment from risks that can be posed by chemicals, enhance the competitiveness and innovation of the chemicals industry, and place greater responsibility on industry for assessing and managing the risks from chemicals and for providing appropriate safety information on substances to their users. This integration may at times have both positive and negative impacts on worker health and safety. For example, LEED credits result in a positive impact on construction worker health when low volatile organic compounds (VOC) adhesives and sealants are used. However, these sealants may negatively impact these same workers because they are reportedly more difficult to use [48].

Recent research has illustrated that LEED certified projects may incur higher injury rates than conventional construction projects [49]. Other research has shown that workers on LEED construction projects are exposed to work at heights, with electrical current, near unstable soils, and near heavy equipment for a greater period of time than on traditional projects [47]. Workers are often exposed to new high risk tasks such as constructing atria, installing green roofs, and installing photovoltaic (PV) panels. Work is under way to suggest modifications to the LEED rating system to include occupational safety and health. Rajendran and Gambatese [49] have developed and validated a Sustainable Construction Safety and Health (SCSH) rating system drawing upon experts from across the U.S. to identify safety and health program elements that are used on construction projects, and assessing those elements to gauge their impact on improving worker safety and health (refer to http://sustainablesafetyandhealth.org/).

### Personal protective equipment: last line of defense

Personal protective equipment is the last line of defense to protect workers from hazardous exposures. Personal protective equipment may be essential in special operations, spills or one-time exposure instances. Other steps of the hierarchy of controls are always preferred, but if necessary, dermal and respiratory protection can reduce worker exposures.

One case study illustrates the downside of using a green alternative retrofit without the use of engineering controls and personal protective equipment. The case study also illustrates that some of the materials or processes that have been labeled as green can actually be harmful to workers; the focus on environmental sustainability sometimes ignores existing or potential worker hazards [20,50-60]. This case study also shows the consequences of not considering workers’ health when making decisions about green alternatives. Ozone-depleting substances, such as chlorofluorohydrocarbons, hydrochlorofluorocarbons, and 1.1.1-trichloroethane, have been replaced by materials, such as 1-bromopropane (1-BP), which is believed to have limited potential to cause ozone depletion [52-54]. This substance has also been used in dry cleaning as a “green” alternative to perchloroethylene, (PERC) which is a known environmental and health hazard [55]. Experimental animal studies provided evidence that 1-BP is a likely reproductive, carcinogenic, and neurological hazard [56-58], which is supported by the onset of neurological and reproductive effects in workers [58-60]. The neurological effects of 1-BP have been documented via a case study of a worker who retrofitted dry-cleaning equipment that traditionally used PERC to accommodate a new solvent containing 1-BP. Following the retrofit process, the worker used the equipment for two days after charging the system during which he experienced significant neurological health effects. Investigation of this event determined that the worker did not wear personal protective equipment during the retrofit or daily operations of the dry-cleaning equipment resulting in exposures to 1-BP.

## Life cycle assessment

A holistic framework in which to consider the relationship between sustainability, green chemistry, and occupational health is the product life cycle [61]. The life cycle includes the supply chain as defined by Handfield and Nichols as encompassing “all activities associated with the flow and transmission of goods through the end user, as well as the associated information flows,” and also activities associated with recycling and end-of-life [62]. Clearly, the principles of green chemistry and sustainability are recognizable as advantageous components of a life cycle approach and analysis. However, life cycle analysis (LCA) has often lacked a sustainability perspective because even though the name implies a cradle to grave view, there are system boundaries in traditional LCA [63]. In short, this means that in an overview of the whole system, all issues that are in conflict with basic sustainability principles have not been taken into account. Consequently, trade-offs, as Ny et al. (2006) observed, between specificity and depth one on hand, and comprehension and applicability on the other, are difficult [63]. Similarly, if workers are not considered in basic sustainability principles, the LCA will be deficient. Workers are integral to all activities from product cradle to grave and have been recognized to some extent by the Global Reporting Initiative (GRI) and the World Bank (WB). Occupational safety and health elements are incorporated in the GR Performance Indicator [9]. The WB Groups Investment Climate Department is supporting research that shows that occupational safety and health issues have been considered in various corporate social responsibility codes of conduct [9]. Nonetheless, the broad consideration of occupational safety and health in sustainability assessments all along the life cycle is lacking. Moreover, research on innovation of new green chemistries appear to be not well funded nor focus on the benefits of worker health and safety [64].

Sustainability, occupational safety and health, and green chemistry can be promoted through the supply chain and ultimately be integral components of life cycle analysis. The triggers to promote these values in supply chains rely in large part on company motivations. Corporate social responsibility agendas need to include this focus and so do international management standards codes of conduct, international framework agreements, and national legislative initiatives.

## Recommendations to enhance the integration of occupational health with green chemistry

Considering the hierarchy of controls can foster the integration of occupational safety and health with green chemistry principles. This integration can promote green chemistry and sustainability while protecting worker health. For a job, process, or chemical to be truly green, it must be safe for the person who performs the work or comes in contact with the substance or product, which means that “the environmental concept of sustainability must be enlarged” [16]. The following recommendations could help maximize occupational health benefits from the innovations informed by green chemistry and sustainability:

1) Consider occupational safety and health in the design and implementation of green chemistry technologies and the selection of alternatives.

Identifying and eliminating hazards to workers at the beginning of any manufacturing or production process design or redesign, including those informed by the principles of green chemistry, affords the opportunity to maximize health gains, as well as environmental benefits and cost savings [9,51,61]. Ultimately, public policies designed to promote green chemistry technologies can protect worker health by including occupational safety and health criteria.

2) Include workers as partners in the implementation of green chemistry innovations.

Many workers lack access to the right kind of hazard information to identify and prioritize chemicals in the workplace*.* Once engaged and informed, workers can suggest work practice changes to reduce exposure and improve productivity. The challenge is how to employ green chemistry in a way that makes it a vehicle for advancing worker safety and health [65].

3) Plan for a transition period toward implementation of green chemistry principles.

It will take time to achieve widespread implementation of green chemistry principles. In the interim, it is crucial to recognize that exploratory approaches and development of the early green products are not necessarily safe for workers. Additional protections are needed, even in green building and production.

4) Develop surveillance approaches to identify potential workplace hazards and assess occupational safety and health hazards that arise through the implementation of green chemistry technologies.

Occupational health surveillance is an essential component of an effective occupational safety and health program [29,66-68]. It involves identifying potentially hazardous practices or exposures in the workplace and assessing the extent to which they can be linked to workers, the effectiveness of controls, and the reliability of exposure measures [67]. Hazard surveillance is a part of occupational health surveillance which also includes medical surveillance. This involves assessing both individual workers for biologic effects or diseases associated with exposure, and also groups of workers for trends of effects or disease.

5) Establish approaches to identify a sentinel event (i.e. injury, illnesses or diseases) in workplaces where green chemistry and sustainability principles are applied.

The importance of identifying sentinel events cannot be overemphasized for emerging technologies and is essential for evaluating control strategies. An occupational sentinel event is defined as a disease, or untimely death, which is occupationally related and whose occurrence may: 1) provide the impetus for epidemiologic or industrial hygiene studies; or 2) may serve as a warning signal indicating that materials substitution, engineering control, personal protection, or medical care may be required [69,70]. Generally, occupational safety and health sentinel events pertain to injury, illnesses or disease for which there is evidence to relate the event to a known occupational hazard. This will be helpful in identifying known hazards in unfamiliar scenarios. When further information is needed to establish the relation of the disease to occupation or industry, a candidate sentinel event may be identified.

## Conclusions

Approaches that utilize green chemistry principles are needed if the U.S. and other nations are going to meet their societies’ needs without compromising the ability of future generations to meet their own needs. These approaches are intertwined with work processes and consequently with workers. Green chemistry and sustainability offer a unique opportunity to improve occupational safety and health through the application of the hierarchy of controls. Ultimately, when worker hazards and risks are considered during design or re-design of processes or products in consideration of green principles, health gains, environmental benefits, and cost savings can be maximized. Public policies designed to promote green chemistry technologies can promote worker health by including occupational safety and health criteria. New and emerging areas such as green nanoscience and green building initiatives such as USGBC’s LEED certification systems offer promising opportunities to utilize green chemistry principles, create sustainability, and protect and promote occupational safety and health. To truly realize all the benefits of sustainability, any hazards to workers that may result from the practical application of green chemistry, green processes, and the manufacture and use of green products should be considered and addressed. Furthering the ties between occupational safety and health and green chemistry could create a synergism that will benefit both endeavors above that which would occur from treating each one separately.

## Abbreviations

1-BP: 1-bromopropane; ACS: American Chemical Society; CO2: Carbon dioxide; EPA: Environmental Protection Agency; LEED: Leadership in Energy and Environmental Design; LCA: Life cycle analysis; NIOSH: National Institute for Occupational Safety and Health; OSH: Occupational safety and health; PERC: Perchloroethylene; PV: Photovoltaic; RCF: Refractory ceramic fibers; REACH: Registration, Evaluation, Authorization and Restriction of Chemicals; SCSH: Sustainable construction safety and health; TSCA: Toxic Substances Control Act; USGBC: U.S. Green Building Council; VOC: Volatile organic compounds.

## Competing interests

The authors declare that they have no competing interests.

## Authors’ contributions

PS: Drafted the original outline for the paper and wrote various sections. LM: Organized the manuscript and contributed to its general development. DH: Contributed to various sections. AO: Provided overall guidance to the development of the manuscript. GD: Contributed examples. TL: Contributed to various sections. CG: Contributed examples. CB: Contributed to various sections. PH: Contributed to various sections. All authors have read and approved the final version.
